# Using Zinc Oxide Nanoparticles to Improve the Color and Berry Quality of Table Grapes Cv. Crimson Seedless

**DOI:** 10.3390/plants10071285

**Published:** 2021-06-24

**Authors:** Mohamed K. Abou El-Nasr, Hussein M. El-Hennawy, Mina S. F. Samaan, Taher A. Salaheldin, Ahmed Abou El-Yazied, Ashraf El-Kereamy

**Affiliations:** 1 Department of Horticulture, Faculty of Agriculture, Ain Shams University, Cairo 11566, Egypt; Mohamed_aboelnasr@agr.asu.edu.eg (M.K.A.E.-N.); T1salah@yahoo.com (H.M.E.-H.); minasamaan@agr.asu.edu.eg (M.S.F.S.); ahmed_abdelhafez2@agr.asu.edu.eg (A.A.E.-Y.); 2 Pharmaceutical Research Institute, Albany College of Pharmacy and Health Sciences, Albany, NY 12144, USA; taher.salaheldin@acphs.edu; 3 Department of Botany and Plant Science, University of California Riverside, Riverside, CA 92507, USA

**Keywords:** Crimson seedless, grape, NPs, ZnO, enzymatic, PAL, SOD, anthocyanin

## Abstract

Producing high-quality table grapes is becoming a challenge in the warmer area of the world due to the global increase in temperature, which negatively affects anthocyanin biosynthesis and other fruit quality attributes. Nanotechnology is a growing field that can be a very useful tool to improve crop productivity and sustainability. The red color is one of the major fruit quality parameters that determine table grape marketability. This study aimed to investigate the role of the zinc element in improving the marketable characteristics of Crimson seedless (*Vitis vinifera L*.) table grape berries i.e., color, firmness, total soluble solids and sugars; besides its role in activating PAL and SOD enzymatic systems. Additionally, this paper investigated the additive advantages of zinc when applied in nanometric form. Five concentrations of zinc oxide nanoparticles, ZnO NPs (0, 25, 50, 100 and 250 ppm), were compared to zinc oxide in mineral form at a concentration of 250 ppm to investigate their effects on the marketable characteristics of Crimson seedless grape cultivar. The treatments were applied as foliar spray on three-year-old Crimson seedless vines grafted on Richter 110 rootstock grown in one of the major table grape production area in Egypt. The experiment was arranged in completely randomized block design and each vine was sprayed with five letters of the solution. The use of the lowest concentration (25 ppm) of ZnO NPs achieved the highest significant enzyme activity (PAL and SOD). Moreover, the T.S.S, sugars and anthocyanin content in berries increased significantly in association of decreasing acidity. On the other hand, the use of a 50 ppm concentration led to an increase in fruit firmness. Collectively, our data showed that 25 ppm of zinc nanoparticles improved PAL and SOD enzymes activity, improved red coloration in table grape and was more effective than the 250 ppm zinc oxide mineral form.

## 1. Introduction

Crimson seedless (*Vitis vinifera L.*) grape cultivar is a late red cultivar and has excellent fruit qualities, good natural flavor, as well as strong and crispy berries [1]. However, under some warmer area, such as in Egypt, the cultivar shows a problem in inadequate coloring development of berries, which is one of the main fruit quality parameters [2] and a key factor in determining fruit marketability [3]. It is observed that 30% or more of the fruit produced by this cultivar may remain on the vine due to insufficient color development [4].

Anthocyanin pigment is responsible for Crimson seedless table grape berry coloration. This pigment is negatively affected by climate and differences between day and night temperature during the veraison stage [5] and it is also affected by the low altitude [6,7]. Various reasons for inferior color development in table grapes have been reported for the conditions prevalent in Egypt, such as high temperature [8,9]) and vigorous growth with dense, shaded canopies [10,11]. The dynamics of anthocyanins and enzyme phenylalanine ammonia-lyase (PAL) activity suggested that PAL is an essential enzyme for their biosynthesis [12].

Many attempts were carried out to solve the inadequate grape coloration; application of the ethylene-releasing compound, 2-chloroethylphosphonic acid (2-CEPA), can hasten the accumulation of anthocyanins in grape skin [13]. Nearly all Crimson Seedless vineyards in California require Ethrel (ethephon) for optimum color development, even though it reduces berry firmness compared to untreated fruit [4]. Additionally, it was reported that abscisic acid (ABA) could increase the anthocyanin content in grape skin and might greatly enhance the color property of grapes [14]. In the same trend, it was noted that there was an increase in the anthocyanin content in Crimson seedless berries following the application with the Gibberellic acid inhibitor, Paclobutrazol [15]. Other phytohormones have been used successfully for this purpose, namely Jasmonic acid [16], salicylic acid [17], 1-naphthaleneacetic acid (1-NAA) [18] and 2,4-dichlorophenoxyacetic acid (2,4-D) [19].

Besides phytohormones, there are other endogenous and exogenous factors that regulate the anthocyanin biosynthesis in grapes. Although sugars are mainly accumulated in the pulp, the total sugar content in berry skin also increases during grape ripening. It has been reported that anthocyanins usually accumulate one week after the massive increase in sugar content, which means the sugars in the skins are closely related to anthocyanin biosynthesis [20]. Micronutrients such as zinc (Zn) had an effective role on grapes. Song et al. (2015 a) [21] stated that Zn treatments enhanced the accumulation of total soluble solids, total phenols, flavonoids, flavanols, tannins and anthocyanins in berry skin, decreasing the concentration of titratable acidity.

Nanotechnology has been recognized as an efficient enhancement in the agricultural field because of its unique physicochemical properties; nanomaterials are increasingly used in agriculture to enhance the biomass of plants because of its small size with a large surface area [22,23]. However, this technique has only recently been used for fruit crops as most studies, such as those reported by [24,25], were conducted on field crops and some vegetable crops. In this regard, foliar spraying of micro-nutrients in nano-form is more suitable than the soil application due to the ease of application, reducing toxicity resulting from the accumulation of micro-nutrients and avoiding fixation in the soil [26].

Zinc Oxide Nanoparticles (ZnO NPs) are nano scaled micro-nutrients which were used in low concentrations and play an important role in plant functions. ZnO NPs enhance the growth characteristics of many plants, including Peanuts [27], Pearl millet [28], Cotton [29], Purslanes [30] and Coffee [31]. ZnO NPs modify the effect of auxin by regulating the tryptophan synthesis and influencing fruit quality [32,33]. Additionally, they act as a co-factor to many enzymes’ activity such as superoxide dismutase (SOD), glutathione peroxidase (GPX) [34,35] and phenylalanine ammonia-lyase (PAL) [36,37].

The goal of this work was to evaluate the influence of ZnO NPs as foliar spray on enzymatic activity and marketable characteristics including color, TSS, sugars and firmness of Crimson seedless table grape.

## 2. Results

### 2.1. Characterization of Zinc Oxide Nanoparticles (ZnO NPs)

**Figure 1:**
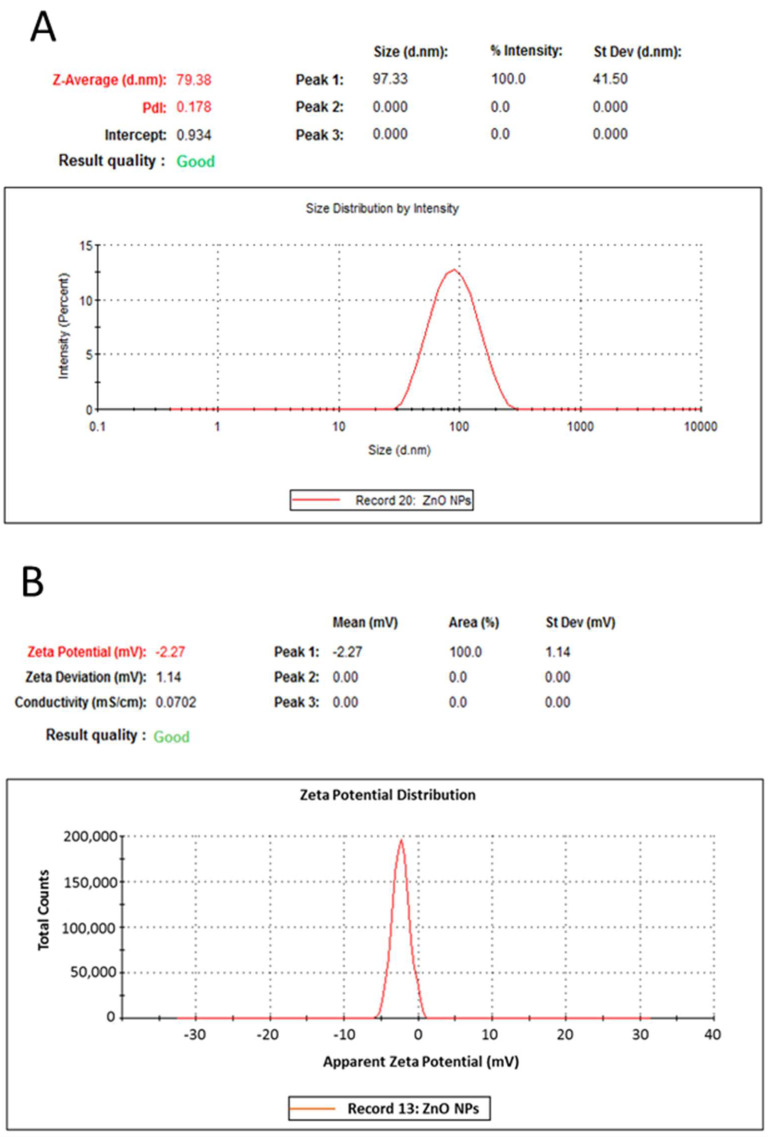
Physicochemical characterization of ZnO nanoparticles. (**A**): particle size distribution measured by DLS. (**B**): Zeta potential measured by ELS.

Zinc oxide nanoparticles were synthesized by co-precipitation method at alkaline pH using urea as a stabilizing and reducing agent as previously described. Dynamic light scattering (DLS) showed the average particle size to be 97.31 nm and zeta potential of −2.27 mV as measured by the electrophoretic light-scattering technique, Figure 1A,B.

**Figure 2:**
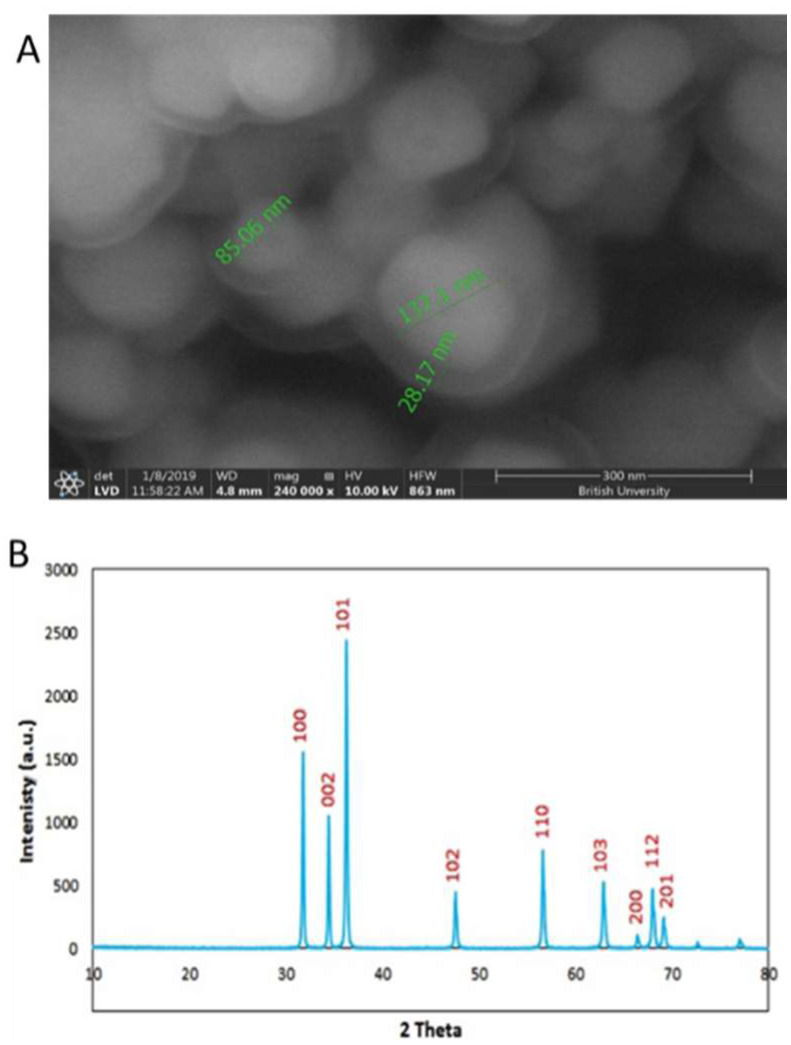
Characterization of ZnO nanoparticles. (**A**): SEM image shows spherical nanoscale (80–130 nm) particles. (**B**): XRD pattern showing the c phase patten of ZnO crystal.

The topographical SEM image showed well-formed spherically shaped 100 nm particles, see Figure 2A, that were in accordance with the DLS measurements. XRD patterns were compared to the standard pattern of ZnO (card #: 01-079-0206), which showed that the diffraction peaks at 2ɵ = 31.7, 34.4, 36.2, 47.5, 65.5, 62.9, 66.3, 68 and 69.1 corresponded to hkl = 100, 002, 101, 102, 110, 103, 200, 112 and 201, respectively which were quite identical to the characteristic peaks of the ZnO crystal, as shown in Figure 2B.

### 2.2. Physiochemical Fruit Properties Evaluation

Data in Table 1 demonstrated that foliar spraying of ZnO NPs significantly affected the studied parameters of crimson seedless grape cultivar during the two seasons. The highest T.S.S. brix values, 18.1 and 19.5, were obtained when the vines were sprayed with 25 ppm of ZnO NPs in both seasons, respectively, while the minimum values of T.S.S., 15 and 15.6, were recorded in berries in the control treatment during the first and second seasons, respectively. Concerning the fruit acidity percentage, the highest percentages were obtained in the berries of untreated vines (control), 0.672 and 0.657, in both seasons, respectively. The lowest acidity percentages, 0.598 and 0.591, were found in the berries of the vines treated with ZnO NPs at 50 ppm in the first and second seasons, respectively. As for the T.S.S./acid ratio, the results revealed that spraying with ZnO NPs at 50 ppm achieved the highest significant percentage, 30.00, in the first season, whereas the second season data favored 25 ppm ZnO NP-treated vines, 32.00. The lowest values (22.7 and 23.8) were found in control vines in the first and second seasons, respectively. Regarding the berries’ firmness, the firmest berries were found in the vines treated with 250 ppm ZnO NPs in the first season (165.6 g/mm); the difference was insignificant when compared to the other treated vines. In the second season, the same treatment produced the firmest berries (264.3 g/mm); the difference was significant compared to the other treated vines, except those of 100 ppm treatment. On the other hand, control vines in both seasons had lower values of berry firmness, 130.6 and 207.9 g/mm, and produced a significant amount of the softest berries.

### 2.3. Chemical Fruit Characteristics

#### 2.3.1. Total Anthocyanin

**Figure 3:**
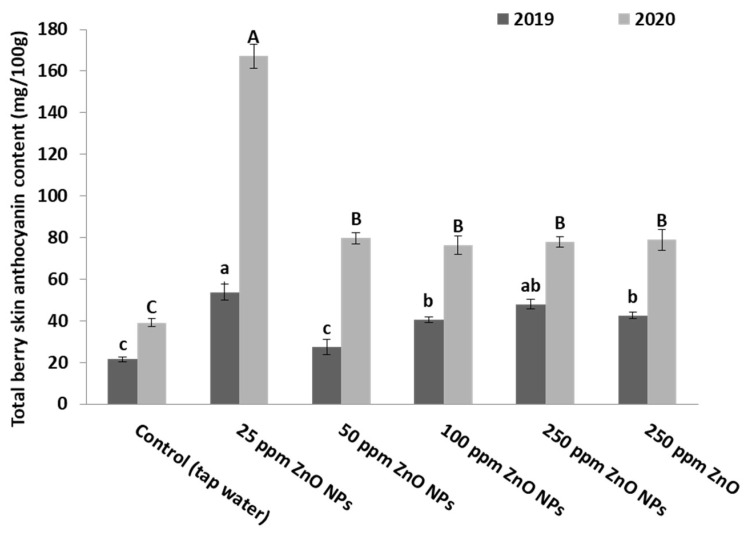
Effect of foliar spraying of ZnO NPs on the total anthocyanin content in the berry skin of Crimson seedless grape cultivar during two seasons: 2019 and 2020. Different letters indicate the differences based on Duncan’s multiple range test.

**Figure 4:**
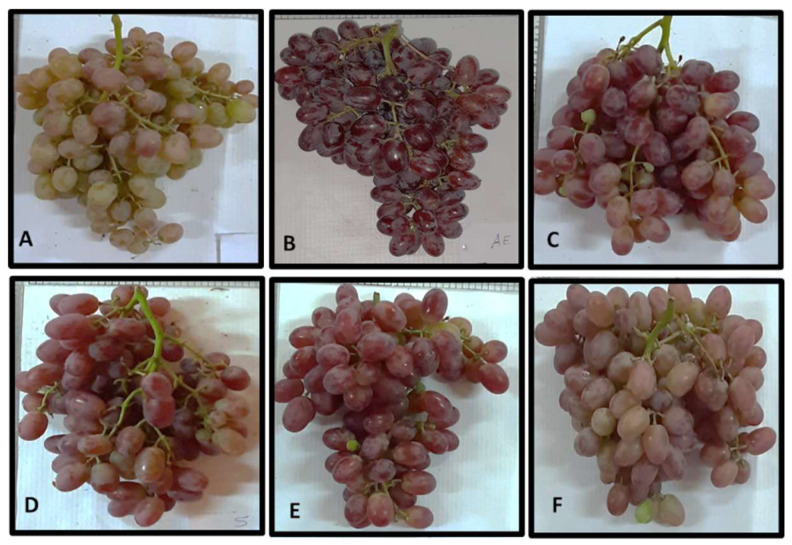
Effect of foliar spraying with ZnO NPs on coloring of Crimson seedless grape clusters. (**A**): water-treated clusters as a control, (**B**–**E**): 25, 50, 100 and 250 ppm ZnO NPs, (**F**): 250 ppm ZnO.

ZnO nanoparticles showed a significant (*p* ˂ 0.05) increase in the anthocyanin content of berries skin in treated vines compared to control vines, as shown in Figure 3. When the vines were treated with 25 ppm of ZnO NPs, anthocyanin content in their berries increased by 150% and 328% compared to the control vines in the first and second seasons, respectively. Figure 4 demonstrates the color differences among the clusters of the treated vines.

#### 2.3.2. Total and Reducing Sugars

**Figure 5:**
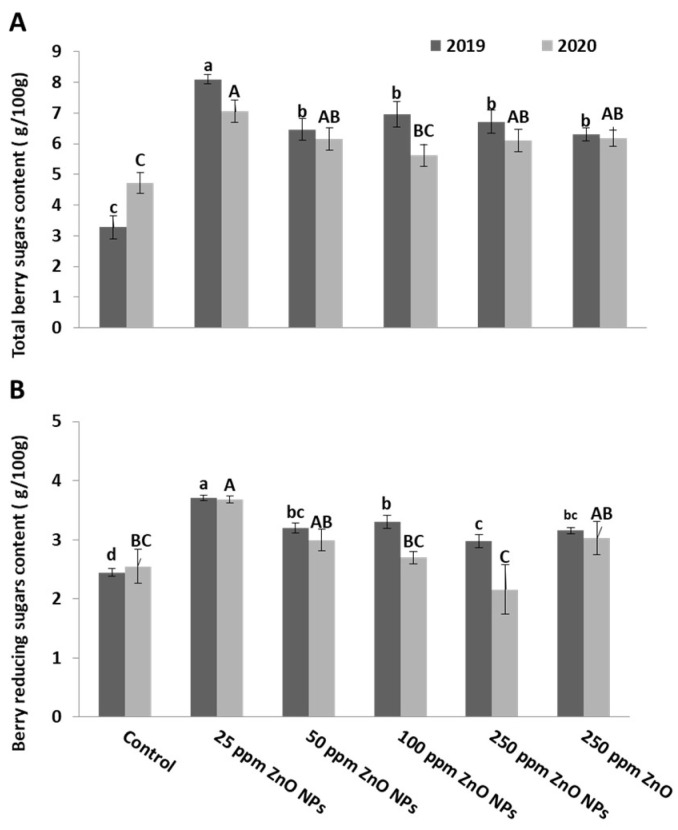
Effect of ZnO NPs foliar spraying on leaf enzymatic activity, (**A**) PAL and (**B**) SOD, of grapevine Crimson seedless cultivar during two seasons: 2019 and 2020. Different letters indicate the differences based on Duncan’s multiple range test.

It is evident that there is an increase in the total and reducing sugar content in berries due to the foliar application of all ZnO nanoparticles concentrations compared to control (Figure 5A,B). The berries of the vines treated with ZnO NPs at 25 ppm achieved the maximum increase in total sugars (8.10 and 7.05) and reducing sugars (3.69 and 3.68) content in both seasons, respectively, compared to ZnO and control treatments.

### 2.4. Enzyme Activity

**Figure 6:**
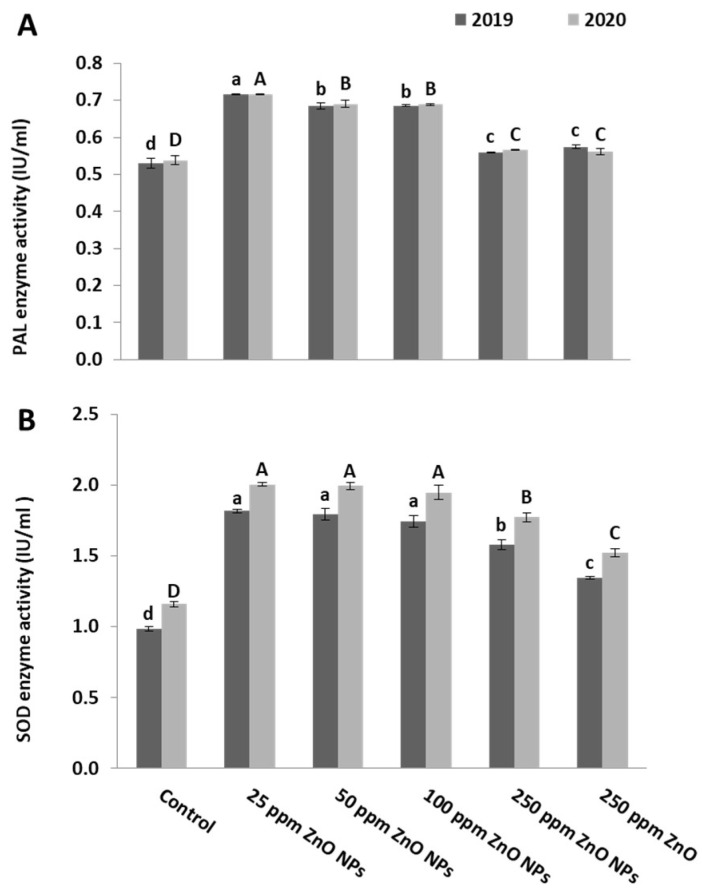
Effect of ZnO NPs foliar spraying on total sugar (**A**) and reducing sugar (**B**) (g/100 g) in berries of Crimson seedless grape cultivar during two seasons: 2019 and 2020. Different letters indicate the differences based on Duncan’s multiple range test.

The level of antioxidant enzymes in mature leaves of the crimson seedless grape cultivar increased by ZnO NPs application in two seasons. An increase in PAL enzyme activity was observed in ZnO NP-treated vines. Similar to previous parameters, the highest activity was observed at 25 ppm ZnO NPs (Figure 6A). Likewise, SOD enzyme activity increased at 25 ppm ZnO NPs (Figure 6B).

## 3. Discussion

As an essential microelement, zinc (Zn) plays a critical role in several plant growth and development functions. Consequently, it significantly affects the growth and quality of agricultural crops. Many studies have showed that the application of Zn can improve the quality of many vegetables and fruits [38,39,40,41]. Nanotechnology provides certain advantages when applying the Zn micronutrient in nanometric form, thanks to its large surface area and intensive surface charge and high resonance of its particles, which includes high absorption efficiency as well as speed uptake and movement inside the plant’s vascular tissues. Data obtained from the current study are in line with these suggestions; no significant difference was observed in the leaves’ Zn content in the 25 ppm ZnO NPs and 250 ppm ZnO during the first year of the study (Figure S2). In the second year, a significant difference was observed in the leaves’ Zn content in the 100 ppm ZnO NPs and 250 ppm ZnO during the second year of the study (Figure S2). Our study investigated the impacts of zinc nanoparticles on fruit quality of Crimson seedless table grape as an important and desired cultivar in Egypt. Zn nanoparticles clearly affected berry firmness, T.S.S., titratable acidity, sugars, anthocyanin content and enzyme activity of PAL and SOD systems.

Our study showed that ZnO NPs foliar spraying enhanced T.S.S., diminished acidity and increased the firmness of Crimson seedless grape cultivar. These effects could be due to the role of Zn in the transference and synthesis of proteins and carbohydrates, as well as the maintenance of the structural stability of cell membranes [33]. Additionally, zinc plays an important role in many biochemical pathways [42]. In this concern, our results were parallel with those of Song et al. [21], who stated that Zn treatments enhanced the accumulation of total soluble solids, total phenols, flavonoids, flavanols, tannins and anthocyanins in berry skin while decreasing the concentration of titratable acidity. Simultaneously, our results also agreed with those of Davarpanah et al. [43], who elucidated that foliar spraying with ZnO NPs led to significant increases in quality of pomegranate fruits, including increases in T.S.S. and decreases in titratable acidity. Additionally, our findings also resembled those of Usha and Singh [44] and Abou-Zaid and Shaaban [45], who reported that zinc improved the total soluble solids and reduced the total acidity of grapes.

ZnO NP application enhances the activity of antioxidant enzymes [46]. In our experiment, ZnO NPs at 25 ppm increased the SOD activity in leaves; the reason of the increase may be due to the role of zinc as a structural and catalytic component to enzymes required for growth and development of plants [47,48]. Moreover, Zn controls the generation and detoxification of free oxygen radicals, which may damage lipids of the membrane and may help to reduce lipid peroxidation rate, since it is a stabilizing and protective component of bio membranes against activated oxygen species [34]. The present observation is supported by the previous work of Kouhi et al. [49], who reported that ZnO NPs in the lowest concentration also enhanced the antioxidant capacity in grapeseed.

The PAL enzyme gives cinnamic acids through the disposal of ammonia from phenylalanine. Cinnamic acids are relatively simple secondary metabolites derived from the shikimic acid pathway in some plants [50]. Foliar spraying of ZnO NPs in our study increased PAL activity in Crimson grape leaves. Our results agreed with the findings of Wadhwa et al. [51], who revealed that Zn acts as a co-factor for PAL enzyme activity. Additionally, Karimzadeh et al. [36] found that the highest activity of the PAL enzyme was found when using 30 ppm nano-ZnO in flax plant.

Anthocyanin synthesis is greatly influenced by temperature. In fact, data presented in Figure S1 showed high maximum temperature peaks in both seasons (2019 and 2020) at veraison stage until harvest. The maximum daily temperature reached 45 °C, which occurred especially frequently in the 2019 season. The minimum temperature (night temperature) was below 20 °C on most nights, which may be favorable for anthocyanin accumulation in the 2020 season compared to the 2019 season, which had night temperatures above 20 °C on most days at the same stage of development, as shown in Figure S1. These interpretations were compatible with what was advocated by Mori et al. [52], who mentioned that a moderate temperature, such as 25 °C, favored anthocyanin biosynthesis, whereas a high temperature, such as 35 °C, is associated with anthocyanin degradation and inhibition of anthocyanin accumulation. Additionally, high night temperature inhibits the gene expression of the anthocyanin biosynthesis genes, CHS, F3H, DFR, ANS and UFGT, at the early stages of ripening and dramatically reduces the activity of UFGT, resulting in poor production of anthocyanins. It is vital to mention that high temperature during the 2019 season, especially night temperature, might delayed the color-based harvest date due to anthocyanin degradation. Moreover, in the 2020 season, the harvest date was earlier because it was cooler than the 2019 season. These findings agree with those of Mori et al. [52], who stated that the C13-labeled anthocyanins were significantly reduced after high temperature treatment, suggesting that the anthocyanin-level was not only influenced by the lower expression of the structure and regulatory genes, but also by the degradation of the previously synthesized anthocyanins. Furthermore, the macroscopic view of Cohen et al. [53] and Ortega-Regules et al. [54] stated that the lowest concentration of anthocyanins in the berries is usually obtained in the warmest years, whereas in the cooler years the grapes produce more anthocyanins.

Anthocyanin biosynthesis occurs in red berry skins through the flavonoid pathway, which starts with the precursor phenylalanine [55]. Foliar application of ZnO NPs at low concentrations enhanced the anthocyanin content in the berries’ skin. Our results were consistent with Song et al. [21], who mentioned that Zn treatments enhanced the accumulation of anthocyanins in berry skin, and Hashemi et al. [56], who explained the role of ZnO nanoparticles in increasing anthocyanin production in soybean. Additionally, Wadhwa et al. [51] mentioned that Zn served as a co-factor for PAL enzyme activity and Medda et al. [12] advocated that Zn played a regulatory enzyme during fruit ripening for flavonoid biosynthesis.

A change of the total and reducing sugar in berries was observed after ZnO NPs treatment, which increased at low concentrations. Raigond et al. [57] agreed with our findings, as they observed that total soluble sugars increased significantly with 500 ppm Zn-NPs treatment in potato plants and reducing sugars in Licorice plants increased with Zn-NPs treatment compared to the control plants [58]. The reason for the increasing sugar content in berries may be due to the role of zinc in carbohydrate metabolism by improving the photosynthesis and sugar transformation [59]. Additionally, Song et al. [21] reported that foliage sprayed zinc sulfate showed promoting effects on the photosynthesis and berry development of vines (the promotion of photosynthesis mainly occurred from the veraison stage to maturation).

Nanomaterials, as a new technology in agriculture, may be met by some anxiety toward its prospective effects on human health. There were various studies that handled the effect of Zn NPs on animals. Long et al. [60] reported that feeding experimental animals with a low dose of ZnO NPs had better effects on growth performance and was beneficial to the urinary system wellness. On the other hand, the biochemical markers in the serum of rabbits showed that there was no toxic effect of ZnO NPs on liver or kidney functions, as the concentration of blood biochemical parameters were in the normal range [61]. In the same context, a previous study revealed that zinc has different ways of protecting the liver from cirrhosis [62]. In conclusion, we provided data on the potential use of nanoparticles as an applied tool to improve red coloration and the marketability of table grapes.

## 4. Materials and Methods

### 4.1. Preparation and Characterization of Zinc Oxide Nanoparticles (ZnO NPs)

Zinc oxide nanoparticles were synthesized by a co-precipitation method using urea reduction of zinc nitrate hexahydrate at alkaline pH [63,64]. Briefly, 50 mL of 1 M urea solution was added to 50 mL of 0.5 M zinc nitrate hexahydrate solution while stirring at 600 rpm at room temperature for 15 min. To this mixture, 10 mL of 1M sodium hydroxide solution was added dropwise under continuous stirring at 800 rpm at 70 °C for 2 h. White suspension of zinc hydroxide (Zn(OH)_2_) nanoparticles then formed. In order to remove unreacted substrates, the suspension was first centrifuged at 40,000× *g* for 30 min, and the precipitate was then washed three times with deionized water (Milli-Q, Millipore, Burlington, MA, USA). Afterward, the precipitate was annealed at 500 °C for 3 h in the muffle furnace (Thermolyne™, Thermo Fisher Scientific, Waltham, MA, USA) to convert zinc hydroxide into white zinc oxide nanoparticles ready for field application after physicochemical characterization.

Particle size distribution and surface charge (Zeta potential) were measured by dynamic light scattering (DLS) and electrophoretic light scattering (ELS) (ZS nano, Malvern-PanAnalytical, Westborough, MA, USA). A total of 10 mg of ZnO nanoparticles were suspended in 10 mL of deionized water using an ultrasonic prob at 80 µm amplitude (Q55, Qsonica LLC., Newtown, CT, USA). Then, 1 mL was transferred to a 2 mL cuvette for size measurement and 1 mL was injected into a zeta potential cell for particle surface charge measurement. ZnO nanoparticles surface morphology was examined by scanning electron microscope (ESEM, Quattro S, Thermo Scientific, Waltham, MA, USA) instrument with acceleration voltage of 5–30 kV. Well-dried samples were carefully sectioned, then fixed on specific grids. The crystallographic phase pattern was identified using an X-ray diffractometer (XRD, X’Pert PRO Malvern-PANalytical, Etten Leur, The Netherlands) operated at 45 kV and 30 mA using filtered Cu Kα radiation (λ = 1.5404 Å) in the 2θ from 5° to 80° and data analysis was performed by high score plus software.

### 4.2. Plant Material and Treatments

This experiment was carried out in El-Ghandour farm, located at Cairo Alex Desert Road K78, Egypt at an altitude of 56 m, in two successive seasons (2019 and 2020). The vines of Crimson seedless grape cultivar grafted on Richter 110 rootstock were three years old, mature and chosen as uniform as possible in terms of growth and vigor. Vines were planted at 2 × 3 m apart in sandy soil under drip irrigation system, trellised by Spanish Parron system and trained by the “four arms” system pruned by the Guyot system. All vines were subjected to the same horticultural practices as recommended according to the farm conditions. The daily maximum and minimum temperature records during the two seasons were obtained by the Central Laboratory for Agricultural Climate—the Egyptian Ministry of Agriculture and Land Reclamation for the experimental site “Cairo-Alexandria Desert Road-K78” in Figure S1.

ZnO NPs were used at different concentrations (0, 25, 50, 100 and 250 ppm) compared to the regular ZnO concentration of 250 ppm. The control treatment (0 ppm) consisted of spraying with tap water only. These foliar treatments were applied three times during the season in April, May and June. Each vine received about 5 L of spraying solution until runoff.

### 4.3. Leaf Zn Content Analysis

Twenty leaves/vine from the middle of tagged shoots were separated, washed with tap water, rinsed with distilled water and dried at 70 °C until a constant weight and then ground to a powdery texture. Half a gram of dry sample was wet digested with 20 mL of 95% H_2_SO4 and 5 mL of 30% H_2_O_2_ until the mixture was clear then filtered, transferred quantitatively and filled to a volume of 50 mL using distilled water [65]. The Zn content of a leaf was determined by using Absorption Spectroscopy according to [66]. Data are presented in Figure S2.

### 4.4. Physiochemical Fruit Properties Evaluation

The total soluble solids were determined in berry juice by manually squeezing berries in a refractometer (PZO-RR13, Warszawa, Poland) with a scale of 0 to 35%. As for the titratable acidity (TA), fruits were cut into pieces to make homogeneous mixture. Next, a 50 g sample was taken then processed for 40 s in a blender. Titratable acidity of the juice was determined manually according to AOAC [67]. Berry firmness was determined by using GÜSS Fruit Texture Analyzer (FTA), the maximum force required was recorded in g/mm.

### 4.5. Enzyme Activity

Phenylalanine ammonia-lyase (PAL) enzyme activity (IU/mL enzyme) was calculated by tracking the increase in the absorbance reading at the wavelength of 290 nm due to the formation of cinnamic acid [68]. In brief, a leaf sample of 1 g was ground in 4 mL extraction buffer (the buffer consisted of 1 M phosphate buffer, pH 7, containing 0.1 mM EDTA and 1% polyvinylpyrrolidone (PVP)). The homogenates were centrifuged under cooling for 15 min. The supernatant was collected and used for the assays of enzymatic activities. The enzyme activity was determined by taking 1 mL of a borate buffer (pH 8.8, 0.1 M) solution then adding 1 mL of 12 mM phenylalanine acid. Finally, 0.8 mL of the enzymatic extract was added.

enzyme activity (IU/mL) = ((ΔA/min)/ε) × (test volume/1000) × 106 × (1/0.8)

where ΔA is the difference between reading absorbance, ε is the extinction coefficient = 9630 L mol^−1^ cm^−1^ and the test volume = 2.8 mL.

Superoxide dismutase (SOD) enzyme activity (IU/mL enzyme) was calculated according to the method of Beyer and Fridovich [69], in which the amount of enzyme required to inhibit Nitro Blue Tetrazolium ‘NBT’ is expressed at 50%. In brief, the 1 g leaf sample was ground in 4 mL extraction buffer (the buffer was prepared using 0.05 M phosphate buffer, pH 7.8, containing 0.1 mM EDTA and 1% polyvinylpyrrolidone (PVP)). The homogenates were centrifuged under cooling for 15 min. The supernatant was collected and used for the assays of enzymatic activities. The reaction was mad by taking 0.1 mL of the enzymatic extract and adding it to 3 mL of the reaction mixture (0.05 M L-methionine, 1 mM NBT, 0.01 M EDTA, 50 mM phosphate buffer (pH 7.8), 0.2 M Riboflavin), after which the tubes were placed under two 15 W fluorescent lamps for 20 minutes to start the reaction. The blank tubes were kept in the dark. The absorbance was recorded at 560 nm.

% of inhibition = ((A control − A sample)/A control) × 100

enzyme activity (IU/mL) = (% of inhibition/50) × (1/0.1)

where A is the absorbance reading.

### 4.6. Fruit Bio-Chemical Components

#### 4.6.1. Total Anthocyanin

The total anthocyanin content was determined according to the method of Connor et al. and Lima et al. [70,71] Briefly, one gram of fresh berries skin was soaked for 24 h in acidic alcohol (1 M HCl/methanol as 15:85 *v*/*v*) to extract the pigment. The samples were then measured using a spectrophotometer at a wavelength of 530 nm. The values were expressed as milligrams cyanidin-3-glucoside (c3g) equivalents per 100 g fresh weight. All determinations were performed in triplicates.

#### 4.6.2. Total and Reducing Sugars

Total and reducing sugars were determined by the iodometric titration method according to Shaffer and Hartmann [72]. One gram of fresh leaves received 15 mL of 95% ethyl alcohol and placed into a water bath for 3 h then cooled, filtered and washed by 80% ethyl alcohol. It was then transferred quantitatively—the tube was filled to a volume of 25 mL using 80% ethyl alcohol.

Total sugars: 15 mL of the filtrated extract was added to 5 mL of HCl 2 M; the previous mixture was heated in a water bath (60 °C) for 30 min and then held until cooled. A drop of methyl red was then added until reaching a light pink color and the beaker was filled up to 50 mL with distilled water. The total sugars were determined by adding 5 mL of beaker contents to 5 mL of Fehling’s solution and heating the mixture until boiling vigorously for 15 min in a water bath. Afterward, it was subjected to running water for 3 min to cool followed by the addition of 2 mL of potassium iodate 2% and 2 mL of sulfuric acid 2 M. Titration was done using sodium thiosulfate solution until the solution had a yellowish-green color, after which drops of starch (blue color) were added until no change in color was observed. The number of milligrams of total dissolved sugars (g/100 g fresh weight) was calculated:

Total sugars (g/100 g fresh weight) = (250/100) × (90/A × 35.67) × (beaker capacity/5) × (B blank − B sample) × (100/sample weight) × (50/15)

where A is the thiosulfate molarity and B is the titration reading.

Reducing sugars: 10 mL of the filtrated extract was placed in a water bath (60 °C) for 15 min, then transferred quantitatively using hot distilled water to a beaker and three drops of phenol indicator were added. Once the solution was titrated using sodium hydroxide 0.2 M, 5 mL of lead acetate was added by dripping. To neutralize the excessive amount of lead acetate, acidic sodium phosphate was added. Finally, the beaker contents were filtered and filled up to 25 mL using distilled water. The reducing sugars were determined by adding 5 mL of beaker contents to 5 mL of Fehling’s solution, after which the mixture was heated until boiling vigorously for 15 min in a water bath. It was then subjected to running water for 3 min to cool, followed by the addition of 2 mL of potassium iodate 2% and 2 mL of sulfuric acid 2 M. Titration was done using a sodium thiosulfate solution until it had a yellowish-green color, after which drops of starch (blue color) were added until the color disappeared. The number of milligrams of total dissolved sugars (g/100g fresh weight) was calculated:

Reducing sugars (g/100 g fresh weight) = (250/100) × (90/A × 35.67) × (beaker capacity/5) × (B blank − B sample) × (sample weight/100)

where A is the thiosulfate molarity and B is the titration reading.

### 4.7. Statistical Analysis

The experiment was arranged in complete randomized block design; each treatment was conducted by five replicates. All statistical analysis of the different traits was performed using the SPSS program software (SPSS, 20). Differences among treatments were tested by Duncan’s Multiple Range test [73].

## 5. Conclusions

From the previously explored results, we can conclude that Crimson seedless grapevines treated with ZnO NPs at a concentration of 25 ppm showed promising responses in terms of leaf enzyme activity as well as the total and reducing sugars in berries. Additionally, anthocyanin accumulation enhanced significantly when vines received the same treatment under a favorable temperature range. This work sheds light on the involvement of the antioxidant enzymes activity in improving table grape berry quality.

## Figures and Tables

**Table 1:** Effect of foliar spraying of ZnO NPs on some physiochemical fruit properties of Crimson seedless grape cultivar during two seasons (2019 and 2020).

Character (Means)	T.S.S (Brix)	Acidity %	T.S.S/Acid Ratio	Firmness (g/mm)	T.S.S (Brix)	Acidity %	T.S.S/Acid Ratio	Firmness (g/mm)
Treatments	First season (2019)				Second season (2020)			
0 ppm (Control)	15.0 ^c^	0.672 ^a^	22.4 ^c^	130.6 ^b^	15.6 ^b^	0.657 ^a^	23.7 ^b^	207.9 ^d^
25 ppm ZnO NPs	18.1 ^a^	0.616 ^ab^	29.5 ^a^	152.8 ^a^	19.5 ^a^	0.613 ^ab^	32.0 ^a^	240.0 ^bc^
50 ppm ZnO NPs	17.7 ^a^	0.598 ^b^	29.7 ^a^	156.9 ^a^	18.6 ^a^	0.591 ^b^	31.7 ^a^	236.0 ^bc^
100 ppm ZnO NPs	17.2 ^a^	0.666 ^a^	25.9 ^b^	160.1 ^a^	18.6 ^a^	0.627 ^ab^	29.9 ^a^	261.1 ^ab^
250 ppm ZnO NPs	17.5 ^a^	0.628 ^ab^	27.9 ^ab^	165.6 ^a^	19.3 ^a^	0.626 ^ab^	30.9 ^a^	264.3 ^a^
250 ppm ZnO	16.3 ^b^	0.616 ^ab^	25.8 ^b^	154.7 ^a^	16.5 ^b^	0.648 ^a^	25.7 ^b^	218.6 ^cd^

Means in each column with the same letter(s) are not significantly different at 5% level. Different letters indicate the differences based on Duncan’s multiple range test.

## Data Availability

All data are contained in this manuscript.

## Supplementary Materials

The following materials are available online at https://www.mdpi.com/article/10.3390/plants10071285/s1, Figure S1: Daily minimum and maximum temperature (A & B) between February to August during the two seasons 2019 and 2020 in the experimental site., Figure S2: Leaves Zinc content of Crimson seedless grapes following different treatments with ZnO and ZnO NPs. Different letters indicate differences based on Duncan’s multiple range test.
