# Supplementary material for: Using Zinc Oxide Nanoparticles to Improve the Color and Berry Quality of Table Grapes Cv. Crimson Seedless
Source: Plants (Basel). 2021 Jun 24;10(7):1285. doi: 10.3390/plants10071285 (PMC8309036; doi:10.3390/plants10071285)
Supplement: Supplementary file 1 [file plants-10-01285-s001.zip › plants-1269432-supplementary.pdf]

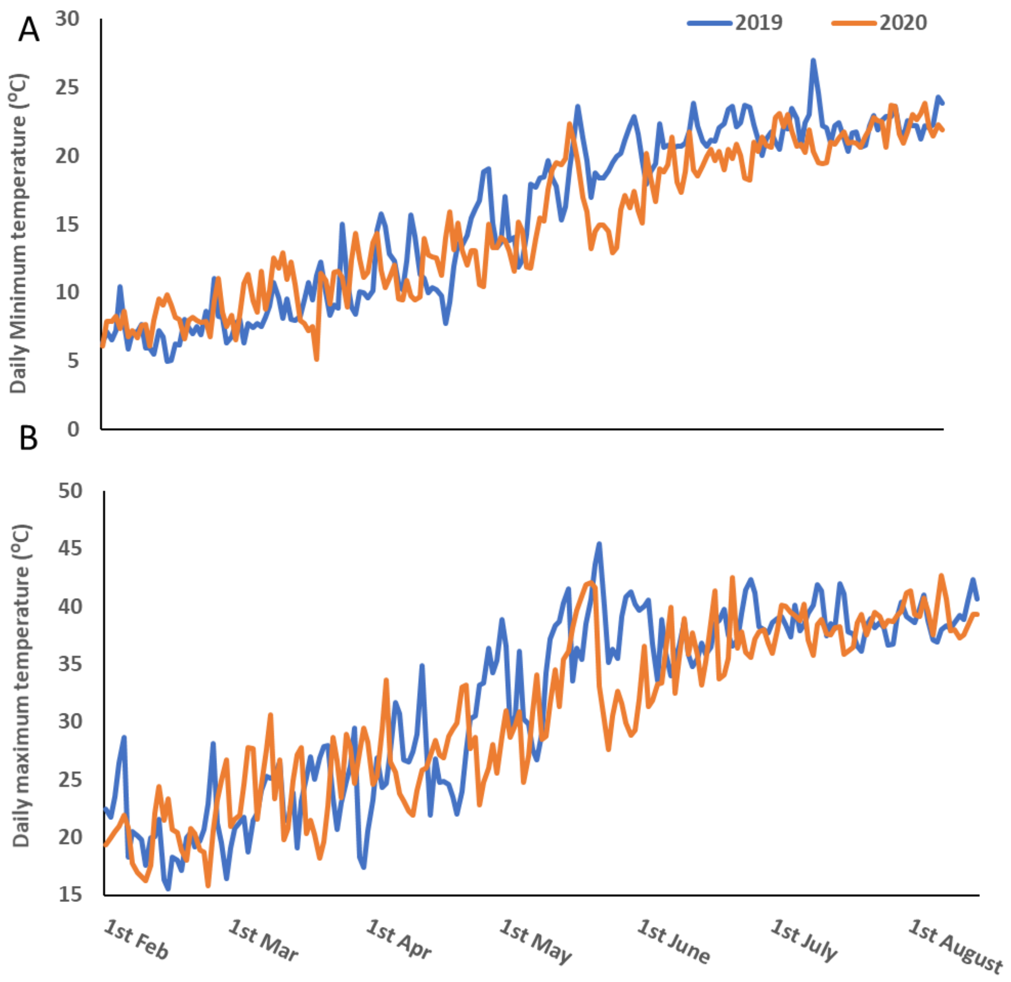

Figure S1. Daily minimum and maximum temperature (A & B) between February to August during the two seasons 2019 and 2020 in the experimental site.

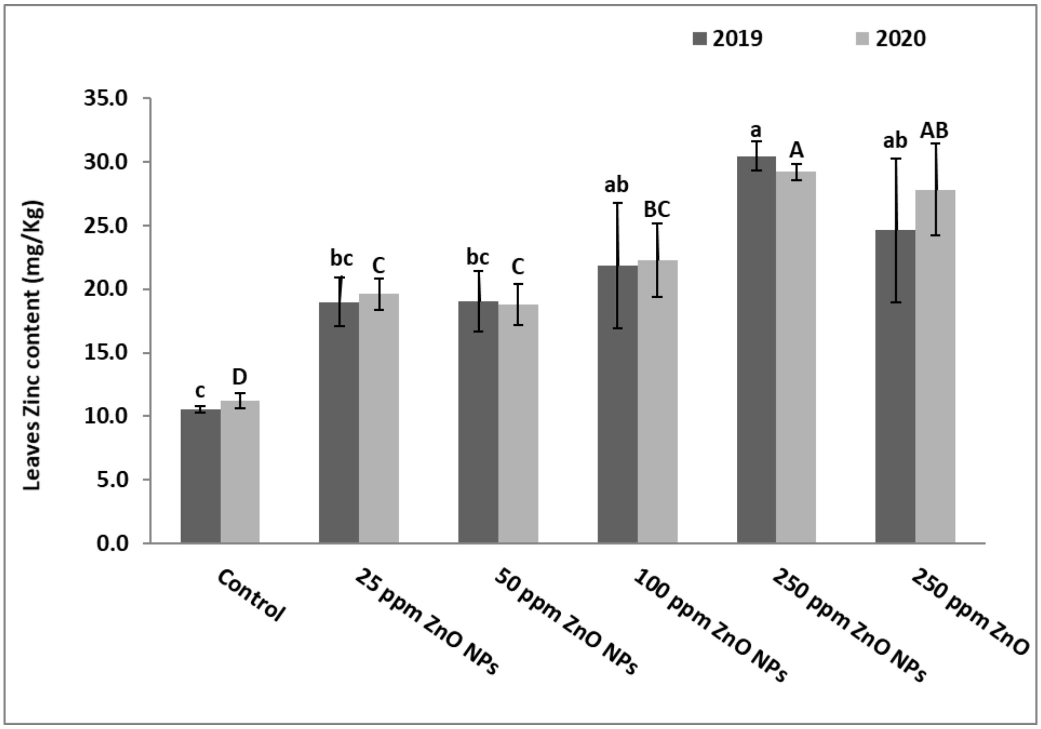

Figure S2. Leaves Zinc content of Crimson seedless grapes following different treatments with ZnO and ZnO NPs. Different letters indicate differences based on Duncan's multiple range test.
